# Discrepant Parent-Adolescent Reports of Parental Monitoring And Their Relations to Cannabis Use Among Justice-Involved Youth

**DOI:** 10.26828/cannabis/2022.02.001

**Published:** 2022-07-11

**Authors:** Lauren Micalizzi, Nazaret Suazo, Claudia Paszek, Lynn Hernández, Kathleen Kemp, Kristina M. Jackson, Anthony Spirito

**Affiliations:** 1 Center for Alcohol & Addiction Studies, Brown University, School of Public Health; 2 Department of Behavioral and Social Sciences, Brown University; 3 Department of Psychiatry and Human Behavior, Warren Alpert Medical School of Brown University; 4 CUNY School of Medicine, The City College of New York; 5 Rhode Island Family Court Mental Health Clinic

**Keywords:** cannabis use, juvenile justice, youth, parental monitoring, sources of parental knowledge

## Abstract

Increased parental monitoring is protective against cannabis use (CU) for justice involved youth, although discrepancies across parent/adolescent reports of monitoring may confer risk. Baseline data were drawn from two randomized clinical trials (152 adolescents; *M*_age_= 15.9; 68% male). Adolescents reported on past 60-day CU and adolescents and parents completed a measure of parental knowledge, parental solicitation, parental control, and child disclosure. Multiple regression models that varied operationalization of discrepancies were performed, in which CU was predicted from each monitoring construct. Inclusion of main effects of parent and adolescent reports improved prediction of CU, particularly parental knowledge and child disclosure. When operationalized categorically, discrepancies improved prediction of CU for parental knowledge. Discrepancies did not improve prediction of CU for the other aspects of parental monitoring. Findings diverge from previous research on adolescent alcohol use; explanations of findings and implications for treatment are discussed.

The prevalence of past-year cannabis use (CU) increased among justice-involved youth (JIY) in recent years (Vaughn et al., 2020) and this trend is expected to continue as the cannabis legalization landscape evolves; as states continue to legalize adult CU, studies show corresponding increases in use among youth who had previous contact with the justice system (Kan et al., 2020). This pattern suggests that less-restrictive policies surrounding cannabis and resulting changes in nationwide perceptions of recreational CU, as well as access to cannabis, may beget increased CU within a group already at risk for CU (Kan et al., 2020). This is concerning because there are negative consequences associated with regular CU among JIY. For example, the presence of a substance use disorder(s) (including cannabis use disorder) in court-involved, non-incarcerated youth was associated with increased risk for general recidivism (Aebi et al., 2021). This evidence highlights the importance of studying and minimizing the risk factors associated with CU among JIY. One of these risk factors is familial context; increased parental monitoring is associated with reduced recidivism and related risks for youth who were previously arrested (Kennedy et al., 2018).

Parental monitoring and knowledge are two of the most frequently evidenced protective familial management strategies for youth substance use (Crouter & Head, 2002). Consequently, targeting parental knowledge and monitoring of teen’s substance use has been a focus in treatment in order to promote and support positive behavior change for adolescents (McGillicuddy & Eliseo-Arras, 2012). It is important to characterize which dimensions of parental monitoring are most protective against substance use to improve and better implement familial interventions, and this begins with thoughtful operationalization of parental monitoring and related aspects of monitoring. *Parental monitoring* reflects a set of discrete parenting behaviors that involve attention to and tracking of the child’s behaviors (Dishion & McMahon, 1998). This definition implies that parents intentionally and actively seek information about their adolescent’s behavior (Crouter & Head, 2002). *Parental knowledge* represents what parents know about their children’s behavior, while *sources of knowledge* represent information acquisition methods—that is, *how* parents come to learn about their child’s behavior (Stattin & Kerr, 2000). Three sources of knowledge have been proposed: *parental solicitation* involves parental garnering of information; *parental control* entails parental imposition of rules on activities; and *child disclosure* is the unprompted reception of information from the child. Evaluating sources of parental knowledge is most relevant if the goal of the research question is to identify discrete parenting behaviors (and thus, active ingredients) that may be targeted through intervention (Micalizzi et al., 2019).

Mounting research across early (Baker et al., 1999), middle (Beck et al., 1999; Mott et al., 1999; Richards et al., 2004), and late adolescence (Barnes et al., 2000; Siebenbruner et al., 2006) shows that greater monitoring and knowledge (particularly child disclosure) are associated with lower alcohol, cigarette, and illicit drug use among general population samples (Abar et al., 2014; Leventhal & Brooks-Gunn, 2000; Li et al., 2000; Micalizzi et al., 2019). Parental monitoring and knowledge may be especially protective among JIY, as research among delinquent youth (who are not necessarily justice involved) highlights this possibility. For example, a meta-analysis of parenting behaviors and delinquency indicated that higher parental monitoring was strongly negatively associated with delinquency (Hoeve et al., 2009). There may also be reciprocal associations between parental knowledge and delinquent behavior, such that low levels of parental knowledge longitudinally predict increased delinquent behavior and that high levels of delinquent behavior predict lower levels of parental knowledge (Laird et al., 2003). More recent studies suggest that the *sources* of parental knowledge, specifically, are a significant factor to consider when predicting problem behavior. Child disclosure of behaviors emerged as a stronger predictor of changes in delinquent behavior over time compared to active parental monitoring (Kerr & Stattin, 2000; Kerr et al., 2010). Thus, the relations between aspects of parental monitoring and problem behaviors are complex, prompting further inquiry into the nature of these associations among JIY.

Parental monitoring tends to be assessed via self-report, which begs the question of who should report on the behavior. Studies that assess parental monitoring reported by both non-JIY adolescents and parents find that parents report higher levels of monitoring than their adolescents (e.g., Abar et al., 2014) and that there is only small or moderate correspondence across reports. This pattern indicates that each member of the dyad may capture different aspects of parental monitoring (Abar et al., 2014) or that they perceive the salience and effectiveness of these aspects of monitoring differently. Discrepant parent/adolescent reports of parental monitoring do not solely represent measurement error; rather, the lack of correspondence across ratings may have predictive value. For example, increased rater discrepancies indicate higher risk for adverse adolescent outcomes, including substance use (Abar et al., 2014; Tremblay Pouliot & Poulin, 2020) and delinquency (De Los Reyes et al., 2010; Ksinan & Vazsonyi, 2016) among general population samples. Research suggests that adolescent reports of monitoring are more strongly predictive of their own substance use than parents’ reports (e.g., Kerr & Stattin, 2000). However, there is also evidence for interactions between parents’ and adolescents’ reports, such that the negative association between child-reported parental knowledge and delinquency was stronger when their mother also reported high parental knowledge (Reynolds et al., 2011). Consequently, discrepant parent-adolescent reports of parental monitoring may have predictive utility with JIY, and more importantly, may reflect underlying mechanisms (e.g., poor communication; Korelitz & Garber, 2016) that should be addressed in an intervention. To date, there is a dearth of research examining discrepancies between reports of parental monitoring and associations with CU, and to our knowledge, this has not been assessed among JIY. Further, parental monitoring and rater discrepancies may have differing associations with CU relative to other substances due to the evolving cannabis landscape.

There are many terms used to characterize discrepancies (e.g., incongruence, inconsistencies, divergence, difference), yet in essence, all refer to the variation in agreement across parent and adolescent ratings (De Los Reyes et al., 2010). While there is clear utility in evaluating discrepancies, care must be taken in operationalizing them. Many researchers test agreement/discrepancy hypotheses using difference scores (i.e., rater 1 [adolescent] minus rater 2 [parent]; Osborne & Lonigan, 2019), yet there are conceptual and statistical limitations to this approach (Laird, 2020; Laird & De Los Reyes, 2013). To this end, a previous study (Abar et al., 2014) tested the utility of different conceptualizations of parent/adolescent discrepancies in reports of parental monitoring and their utility in predicting alcohol use over time. The researchers found that adolescent reports were better predictors of alcohol use than parent reports, yet greater discrepancies were uniquely associated with higher likelihood of alcohol use.

Building off this foundational work (Abar et al., 2014; Laird, 2020; Laird & De Los Reyes, 2013), the current study examines the utility of various operationalizations of parent/adolescent discrepancies of parental knowledge and sources of parental knowledge in predicting past 60-day cannabis use among JIY. It was hypothesized that: 1) for both adolescent and parent reports of parental monitoring, increased monitoring would be associated with less frequent CU; 2) there would be modest correspondence across parent and adolescent reports of all aspects of parental monitoring; 3) parents would report higher levels of parental knowledge, parental control, parental solicitation, and child disclosure relative to adolescents; 4) consistent with Abar et al., 2014, the operationalization of discrepancies based on categorical groupings (i.e., categorical groupings based on adolescent report [high/low] and parent report [high/low]) would be most predictive of CU; and 5) adolescent reports of child disclosure would be most predictive of adolescent CU.

Due to the disproportionate involvement of youth of color in the juvenile justice system, as well as in the present study samples, it is important to probe potentially differing effects of parental monitoring on substance use among youth from different racial and ethnic backgrounds. Although there are some mixed findings (e.g., Smith & Krohn, 1995), most studies find that parental monitoring is protective against deviant behaviors among Hispanic/Latine youth (e.g., Caldwell, 2006; Forehand et al., 1997). This suggests that lower correspondence in reports of parental monitoring across Hispanic/Latine families may be a strong indicator of risky adolescent substance use, but to our knowledge, this has not been explored. Additionally, there is some evidence for racial differences in parental monitoring (e.g., Blustein et al., 2015; Latendresse, Ye, Chung, Hipwell, & Sartor, 2017), yet there is mixed evidence for racial/ethnic differences related to the protective effect of parental monitoring (e.g., Bohnert et al., 2009; Latendresse et al., 2017). To our knowledge, this has not been assessed for CU. Consequently, we explored ethnic and racial differences in discrepancies in exploratory analyses.

## METHODS

### Participants and Procedure

Baseline data (i.e., prior to the intervention) were drawn from two randomized clinical trials (RCT) involving cannabis-using court-involved, non-incarcerated youth (*N*=152) and a participating caregiver. Youth involved in these studies were in the diversion stage of their involvement with the juvenile justice system, often having a first-time offense or low-level second time offense. Participants were eligible if they were English and/or Spanish speakers.

Study 1: 83 youth (73.49% male; *M*_age_=15.93 years) who reported past-year CU at intake to a Family Court in the Northeast United States, were 18 years of age or younger, and lived at home with a parent or guardian were recruited for a study of the effectiveness of a brief motivational interview plus parenting intervention to reduce youth CU (Kemp et al. , 2022). Participants were referred for status (12.64%) and delinquent (87.36%) offenses. Youth in study 1 self-reported race as: 49.37% White, 24.05% Black or African American, 2.53% Native Hawaiian or Other Pacific Islander, 1.27% American Indian or Alaskan Native and 1.27% Asian, 10.13% Other, 11.39% multiracial; 55% identified as Hispanic/Latine. Parents in study 1 (*n*=80; 74.07% female; *M*_age_=41.69) self-reported race as: 17.72% Non-Hispanic Black or African American, 6.33% Hispanic Black; 12.66% Hispanic White, 48.10% Non-Hispanic White, 1.27% American Indian or Alaskan Native, 1.27% Asian, 10.13% Other, and 2.53% preferred not to answer. Caregivers included: biological parents (88%), step parents (4%), adoptive parents (4%) relative guardians (2%) and non-relative guardians (2%).

Study 2: 69 youth (60.87% male; *M*_age_=15.82) who were between 13 and 18 years of age, lived at home with parent or legal guardian, used cannabis at least three times in the prior 90 days, and had a history of truancy in the past school year were recruited for a study evaluating the feasibility, acceptability, and preliminary efficacy of motivational enhancement therapy plus the Family Check-Up for parents; see Spirito et al. (2018) for more information. Participants had histories of truancy and thus were referred for status offenses. Youth in study 2 self-reported race as: 40.58% non-Hispanic White, 13.04% Hispanic White, 18.8% non-Hispanic Black or African American, 4.35% Hispanic Black, and 23.19% Multiracial. Parents in study 2 (*n*=69; 91.3% female; *M*_age_=42.1) self-reported race as: 47.83% non-Hispanic White, 17.39% Hispanic White, 17.39% non-Hispanic Black or African American, 4.35% Hispanic Black, 11.59% multiracial, and 1.45% other. All caregivers were biological parents; 91% of adolescents lived with the participating biological parent and 9% lived with their other biological parent.

These studies were approved by Brown University and/or Lifespan Hospital IRBs.

### Measures

#### Demographics.

Youth and caregivers reported their age, sex, race, and ethnicity.

#### Cannabis use.

The Timeline Followback (TLFB; Dennis et al., 2004; Sobell & Sobell, 1992) covered the number of days of cannabis use over the 60 days prior to the baseline assessment.

#### Parent Monitoring Questionnaire

(PMQ; Stattin & Kerr, 2000), also referred to as Sources of Parental Knowledge, is a 24-item youth and parent report measure designed to assess parental knowledge as well as three sources of parental knowledge (child disclosure, parental solicitation, parental control). Adolescents reported the proportion of time they experience monitoring strategies on a Likert scale (ranging from 1= “No, never [0%]” to 5= “Yes, always [100%]”). Scale scores were computed by reverse scoring items where necessary and averaging relevant items.

Parents and adolescents responded to nine items about parents’ monitoring (i.e., parental knowledge). An example item from the adolescent version includes, “How often do your parents know what you do during your free time?” Parent items reflected identical content but were phrased from the parent’s perspective. Cronbach’s α teen= .84, parent=.86. *Child disclosure* (5 items) evaluated adolescents’ spontaneous disclosure of information about daily activities (e.g., “If you are out at night, when you get home, do you tell what you have done that evening?”; parent items were worded from their perspective); Cronbach’s α teen=.90, parent=.79. *Parental solicitation* (5 items) tapped parental garnering of information about their adolescents’ behavior (e.g., “In the last month, have your parents talked with the parents of your friends?”); Cronbach’s α teen= .74, parent=.77. *Parental control* (5 items) evaluated parental rules and restrictions to control and gain information about their child (e.g., “Do you need to have your parents’ permission to stay out late on a weekday evening?”); Cronbach’s α child=.88, parent=.82.

#### Data Analysis Plan

Associations among parent and child reports of each monitoring-related construct (parental knowledge, parental control, parental solicitation, and child disclosure) were first evaluated using bivariate correlational analyses. Parent and adolescent means for each aspect of monitoring were then compared using dependent samples *t* tests. Next, following Abar et al. (2014), a series of hierarchical multiple regressions were performed. For each aspect of monitoring, four models reflecting different methods to measure parent/adolescent discrepancies were run. In all models, frequency of past 60-day cannabis use was the dependent variable. Sex and age were included as predictors in Step 1 for each model (i.e., Model 0).

In Model 1, main effects of parent and adolescent reports of parental monitoring were entered in Step 2. We took two approaches to measuring discrepancy: forming an interaction between adolescent and parent report (Model 2) and creating a categorical variable based on child and parent report (high/low; Model 3). In Model 2, parent and adolescent reports were standardized and multiplied to create a two-way interaction term and the main effects and interaction term were entered in Step 2. Following Laird, 2020, if the interaction terms were significant, polynomial equations were added in a subsequent step. For Model 3, four groups were created based on mean splits for each aspect of monitoring: 1) low parent/low adolescent (i.e., if both the parent and adolescent fell below the mean on each aspect of parental monitoring); 2) high parent/low adolescent; 3) low parent/high adolescent; and 4) high parent/high adolescent. Three dummy codes were created to reflect these groups and were entered in Step 2 for Model 3; low/low served as the reference group.

Residual diagnostics were performed using the DHARMa package in R (Hartig, 2021) to inform selection of model distributions. Based on these results, a count model with a quasipoisson distribution was used for all models with the exception of one model (Model 2 for parental control), which was modeled using the negative binomial distribution. Incidence rate ratio (IRR) values are reported, which reflect changes in incidence rate for every 1 unit increase in the predictor variable. A value of 1 indicates that there are no changes in the incidence rate of CU for every 1 unit increase in the predictor variable. Values greater than 1 indicate that the incidence rate for CU increases with higher values of the predictor variable, while values less than 1 indicate that the incidence for CU decreases with higher values of the predictor variable. If the confidence interval includes 1, the parameter is not significant.

Goodness of model fit was assessed using chi-square (χ2) difference tests. The relative fit of the expanded model was determined by the log-likelihood difference between an expanded model and a nested model and corresponding change in degrees of freedom (Δ*df*). A significant difference test indicates that the extended model fits significantly better than the nested model. For all monitoring-related constructs, Models 1 (i.e., the main effects model), 2 (i.e., the main effects plus interaction term model), and 3 (i.e., the categorical groups model) were compared to Model 0 (i.e., model included age and sex as predictors of CU). Model 2 was then compared to Model 1. Models 1 and 3 are not nested and thus cannot be compared using formal difference tests. However, we evaluated AIC values to compare fit across non-nested models.

## RESULTS

Baseline differences between the two samples were found on two measures of monitoring; in study 1, youth reported significantly higher levels of child disclosure (*t*[150]=4.05, *p*<.001) and parental knowledge (*t*[150]=3.47, *p*<.001) relative to youth in study 2. There were no significant differences across groups for CU. Descriptive statistics and correlation coefficients are presented in Table 1. On average, adolescents used cannabis on approximately 24 of the prior 60 days (range=0–60, skew=0.52, kurtosis=−1.16). CU was negatively associated with parent and adolescent reports of all aspects of monitoring. There was low-to-moderate correspondence (range=.24, .42) across parent and teen ratings for all monitoring variables. Parent reports of each monitoring-related construct were significantly and positively associated with one another (range=.31, .66), and the same pattern emerged for adolescents (range=.44, .67). Results of the dependent samples *t*-tests indicate that parent ratings of solicitation (*t*[151]=5.82, *p*<.001) and control (*t*[151]=8.40 *p*<.001) were significantly higher than adolescents’ ratings of the same subscales; there were no significant reporter differences for knowledge and disclosure.

### ABC

#### Parental Knowledge.

Results from the model fit comparisons are reported in Table 2. Models 1 and 3 (i.e., the main effects model and the categorical model) fit significantly better than Model 0. Model 2 did not significantly improve explanation of CU relative to Model 1, indicating that the interaction did not aid in the prediction of CU beyond the main effects. Model 3 was the best fitting model, but similar AIC values across Models 1 and 3 indicated comparable fit of these expanded models relative to the base model. Parameter estimates from the hierarchical regression models for parental knowledge are presented in Table 3. In Model 1, adolescents’ reports of parental knowledge were negatively related to CU; parent reports did not significantly predict use. In Model 2, the main effect of adolescent report remained significant and negative, while the interaction term between parents’ reports and adolescents’ reports was not a significant predictor of CU. Because the interaction term was not significant, polynomial interactions were not probed. In Model 3, all groups (i.e., high parent—low adolescent, low parent—high adolescent, and high parent—high adolescent) were associated with less frequent CU relative to the reference group (low parent—low adolescent).

#### Parental solicitation.

Model 1 was the only extended models that improved prediction of CU relative to Model 0, yet in Model 1, neither main effect of adolescents’ or parents’ reports of parental solicitation were significantly associated with CU (see Table 4). Neither the main effects nor the interaction term was significant in Model 2. In Model 3, membership in the high parent— high adolescent group was associated with less frequent CU relative to the low-low group.

#### Parental control.

As with parental solicitation, Model 1 improved fit relative to Model 0. Results from Model 1 (see Table 5) indicated that neither adolescents’ nor parents’ reports of parental control were associated with less frequent CU. Neither the main effects nor the interaction term was significant in Model 2 and none of the mean-split groups were significant predictors of CU in Model 3.

#### Child disclosure.

Models 1 and 2 fit significantly better than Model 0 (see Table 6). Model 3 did not improve fit relative to Model 0, and Model 2 did not improve fit relative to Model 1, indicating that Model 1 was the best fitting model for child disclosure. Adolescent reports of increased child disclosure were associated with less frequent CU in Model 1. This parameter remained significant in Model 2, though the interaction term was not significant and fit for Model 2 was not superior to the fit for Model 1. As with parental knowledge, membership in the high parent—high adolescent group was protective against CU, relative to the low-low group.

### Sensitivity Analyses for Racial and Ethnic Differences in CU

Exploratory sensitivity analyses were conducted to examine if there were mean differences in CU across adolescents identifying as Black or African American vs. non-Black or African American in our sample and across adolescents identifying as Hispanic/Latine vs. non-Hispanic/Latine. The analyses did not reveal any differences for Black or African American vs. non-Black or African American (*F*(1, 150) = [0.31], *p* = 0.58) or Hispanic/Latine vs. non-Hispanic/Latine (*F*(1, 150) = [0.02], *p* = 0.88). Base rates were too low to explore moderation of monitoring and CU associations by racial/ethnic group.

## DISCUSSION

The goal of this paper was to determine if discrepancies are a useful tool for predicting CU among JIY, and if so, how best to operationalize discrepancies among this population. Results indicated that increased parental monitoring was associated with less frequent CU. Results from the regression models indicated that increased adolescent reports of parental knowledge child disclosure most consistently predicted less frequent CU. Operationalizing discrepancies using a categorical approach best predicted parental knowledge, but overall, the discrepancy models fit similarly to the main effects models, suggesting that the inclusion of discrepancies—regardless of how operationalized—did not aid in the prediction of CU beyond the main effects of parent and adolescent report of parental monitoring.

Similar to Abar and colleagues (2014), adolescent reports of parental knowledge were negatively associated with CU. As such, encouraging parents to participate in active monitoring and seeking knowledge of teen whereabouts and interests may create an environment that protects against CU. Further, parental knowledge may be protective against CU because among families in which parents are more knowledgeable about their adolescents’ activities, this may reflect a better parent/child relationship quality; this should be assessed in future research with JIY.

For parental control, adolescent reports of parental control were not significantly associated with CU. This finding diverges from previous work demonstrating that increased parental control is moderately associated with lower levels of problem behavior in early adolescence (Fletcher et al., 2004; Gray & Steinberg, 1999). However, this finding is consistent with more recent literature demonstrating inconsistent results regarding these associations (Keijsers et al., 2010). It is important to underscore that these studies were conducted in community samples; the JIY sample assessed here is likely to face punitive repercussions as a result of continued problem behavior and parental control may be more challenging and may not be an effective tool in decreasing CU among this group. Parents of JIY who exert control as a means to decrease problem behavior may find that this is not an effective approach because parental control is best exerted within the context of a highly supportive parent/adolescent relationship (Micalizzi et al., 2019) and involvement in the juvenile justice system and/or familial disruption that leads to risk behaviors may upset the relational balance within the home. As such, parental control may become an effective monitoring technique if the quality of the parent/adolescent relationship is positive.

Previous research has yielded mixed results as to effectiveness of parental solicitation as a source of knowledge. In line with the current findings, other studies indicate that parental solicitation alone is not sufficiently associated with decreases in problem behavior among adolescents (Keijsers et al., 2010). Laird & colleagues (2010) demonstrated that situational factors, particularly more unsupervised time alone, may impact the relation between parental solicitation and problem behavior, whereby adolescents’ perception of increased solicitation was associated with lower levels of problem behavior.

As has been seen in other studies (e.g., Kerr & Stattin, 2000), adolescent disclosure of their CU also protected against CU. Youth’s open disclosure of their own activities may be most predictive of their CU as this may reflect a more general open communication with parents which has been shown to be related to reduced adolescent substance use (Bertrand et al., 2013). With regard to the pattern of the main effects findings as a whole, it is not surprising that adolescent reports of the different aspects of parental monitoring were most consistently associated with their own CU when compared to parent reports. These findings add to a mounting research base (e.g., Abar et al., 2014) demonstrating this pattern of findings, yet the current study is novel in its extension of these conclusions to JIY and to CU. These results highlight that adolescent reports of parental monitoring are most predictive of their own behavior, and this pattern holds across non-JIY and JIY, as well as across substances (i.e., alcohol in Abar et al., 2014 vs. cannabis in the current study).

Contrary to our predictions, discrepancies did not aid in the prediction of CU beyond the inclusion of parent and adolescent reports for parental solicitation, parental control, and child disclosure. Previous research has demonstrated that cross-rater agreement of parental monitoring is associated with reduced adolescent substance use (e.g., Abar et al., 2014). Specifically, the categorical models (i.e., mean split models) in which parents and adolescents both reported above average monitoring (i.e., the high/high group) tended to be least likely to engage in alcohol use in previous work (Abar et al., 2014). The high/high group was consistently the most protective group for parental knowledge, parental solicitation, and child disclosure. Though the categorical model was the best fitting discrepancy model for parental knowledge, the discrepancy model was only marginally better than the main effects model. The finding that discrepancies did not universally aid in the prediction of CU may have emerged for two reasons: the nature of the study sample and the substance under investigation. First, with respect to the sample: discrepancies across parents and teens in their reports of monitoring may not be indicative of CU risk for JIY relative to non-JIY adolescents. Parent/adolescent reports of monitoring may be more aligned because as families engage with the court system, parents may become aware that they no longer have control of their child’s friend groups or their comings/goings and honestly admit lack of control. Alternatively, parent and adolescent reports may be misaligned, but the misalignment is not predictive of the adolescent’s CU. This may be the case because the correspondence across parents and adolescents is less protective for JIY and presumably already face challenges in the parent/adolescent dynamic as a result. Second: discrepancy findings may be substance specific. Previous research has primarily focused on adolescent alcohol use (e.g., Abar et al., 2014), and thus the replication of these findings to cannabis using non-JIY-involved teens is essential to parse this pattern of results. Further, it is possible that discrepancies may be related to negative consequences of CU and this should be addressed in future research.

The role of the family among Hispanic/Latine youth is well recognized, such that the structure and function of the family may play serve both protective and exacerbating roles in adolescent substance use (Wagner et al., 2010). There were no significant differences in CU across different racial and ethnic groups in the current study. Though our samples are more racially and ethnically diverse than many studies, base rates were not sufficient to test for moderation of parental monitoring on CU by race or ethnicity; future research should probe the extent to which race and ethnicity moderate the effects of parental monitoring on cannabis use, particularly among justice involved samples.

The present study is the first to examine discrepancies in parental knowledge and sources of knowledge among cannabis using JIY. This is also a relatively large JIY sample. This study benefited from the application of multiple methods to operationalize discrepancies and, to our knowledge, this study is the first to assess CU outcomes using this methodological framework. As such, these methodological and practical strengths highlight the relevance and application to JIY. Several limitations should also be noted. First, parental relationship quality was not assessed, and as a result it was not possible to evaluate if particular monitoring strategies were more or less effective based on the context of the quality of the parent/adolescent relationship. Second, all data were collected by self-report, as is the case with most of the literature, so findings may not apply if collected via interviewer or observer ratings. Third, participants were recruited from a single state in the Northeast United States and replication work should be performed on a more geographically representative sample. Fourth, these data are cross-sectional and we hypothesize that parental monitoring is the predictor of CU based on previous research, but the opposite order of effects is also reasonable. Future observational research should address the question of ordering. Finally, all participating families agreed to participate in in a family-focused intervention to reduce adolescent CU which may limit generalizability to other youth involved in the justice system.

In conclusion, these findings suggest that parental knowledge and child disclosure may be protective for JIY adolescents. Generally, discrepancies, regardless of operationalization, did not improve prediction of CU. Higher parental knowledge and child disclosure are two aspects of parental monitoring that may reflect great communication and relationship quality, which may facilitate the effectiveness of parental monitoring techniques and should be a focus on intervention programs.

## Figures and Tables

**Table 1.** Means (Standard Deviations) and Correlation Coefficients on Cannabis Use and Monitoring Subscales

	1	2	3	4	5	6	7	8	9
1. Cannabis Use	24.39 (21.07)								
2. Knowledge (P)	−.17*	3.56 (0.76)							
3. Knowledge (A)	−.21*	**.42** ***	3.43 (0.84)						
4. Solicitation (P)	−.23**	.56***	.29***	3.37 (0.88)					
5. Solicitation (A)	−.18*	.26**	.56***	**.24** **	2.84 (0.92)				
6. Control (P)	−.21*	.38***	.20*	.37***	.11	4.48 (0.78)			
7. Control (A)	−.24**	.34***	.59***	.19*	.48***	**.41** ***	3.71 (1.19)		
8. Disclosure (P)	−.16*	.66***	.33***	.50***	.21*	.31***	.19*	3.23 (0.86)	
9. Disclosure (A)	−.18*	.40***	.67***	.17*	.54***	.09	.44***	**.33** ***	3.22 (0.85)

*Note*. P=parent report; A=adolescent report. Bolded cells depict the correlation between adolescents’ reports and parents’ reports on the same aspect of monitoring.

* *p* < 0.05,

** *p* < 0.01,

*** *p* < 0.001.

**Table 2.** Model Fit Comparisons

Model	Parental Knowledge			Parental Solicitation			Parental Control			Child Disclosure		
	AIC	Δx^2^ (Δ*df*)	*P*	AIC	Δx^2^ (Δ*df*)	*P*	AIC	Δx^2^ (Δ*df*)	*P*	AIC	Ax2 (Δ*df*)	*P*
Model 0	1276.5	—	—	1276.5	—	—	1276.5	—	—	1265.4	—	—
Model 1 (vs. Model 0)	1274.3	6.17 (2)	.04	**1273.9**	**6.60 (2)**	**.04**	**1274.2**	**6.30 (2)**	**.04**	**1260.2**	**9.20 (2)**	**.01**
Model 2 (vs. Model 0)	1276.3	6.20 (3)	.10	1275.8	6.71 (3)	.08	1276.7	5.76 (3)	.12	1262.1	9.35 (3)	.03
Model 3 (vs. Model 0)	**1271.6**	**10.91 (3)**	**.01**	1275.2	7.26 (3)	.06	1276.5	6.00 (3)	.11	1264.5	6.93 (3)	.07
Model 2 (vs. Model 1)	1276.3	0.03 (1)	.86	1275.8	0.10 (1)	.74	1276.7	0 (1)	1.0	1262.1	0.14 (1)	.71

*Note*. Model 0 = base model (age and sex); Model 1 = main effects of adolescent and parent reports; Model 2 = main effects of adolescent and parent reports plus interaction term; Model 3 = mean-split groups. AIC=Akaike’s Information Criterion; Δχ2 = log-likelihood (not reported) difference between nested and expanded model; Δ*df* = change in degrees of freedom of expanded model relative to nested model. Bolded models were the best fitting models for each aspect of parental monitoring.

**Table 3.** Coefficients and Incidence Rate Ratios from Hierarchical Regressions for Parental Knowledge

	*b*	SE	*p*	IRR (*b*) (95 % CI)	β (95 % CI)	IRR (β) (95 % CI)
Model 0						
Age	0.11	0.06	0.06	1.11 (1.00, 1.25)	0.27 (−0.01, 0.54)	1.31 (0.99, 1.72)
Sex (0=male)	0.05	0.15	0.72	1.05 (0.79, 1.40)	0.05 (−0.22, 0.32)	1.05 (0.80, 1.37)
Step 2 Model 1						
Age	0.09	(0.06)	0.10	1.10 (0.98, 1.22)	0.23 (−0.04, 0.50)	1.26 (0.96, 1.64)
Sex (0=male)	0.003	(0.15)	0.98	1.00 (0.75, 1.34)	<.001 (−0.27, 0.27)	1.00 (0.77, 1.31)
Adolescent Reports	−**0.17**	**(0.09)**	**0.04**	**0.84 (0.71, 0.99)**	−**0.29 (**−**0.58,** −**0.01)**	**0.75 (0.56, 0.99)**
Parent Reports	−0.06	(0.09)	0.51	0.94 (0.79, 1.13)	−0.09 (−0.37, 0.18)	0.91 (0.69, 1.20)
Step 2 Model 2						
Age	0.09	(0.06)	0.10	1.10 (0.98, 1.22)	0.23 (−0.04, 0.49)	1.25 (0.96, 1.64)
Sex (0=male)	0.01	(0.15)	0.97	1.01 (0.75, 1.34)	<.001 (−0.27, 0.28)	1.01 (0.77, 1.32)
Adolescent Reports	−**0.17**	**(0.09)**	**0.04**	**0.84 (0.71, 0.99)**	−**0.29 (**−**0.58,** −**0.01)**	**0.75 (0.56, 0.99)**
Parent Reports	−0.06	(0.09)	0.49	0.94 (0.78, 1.13)	−0.10 (−0.38, 0.18)	0.91 (0.68, 1.20)
Adolescent*Parent	−0.02	(0.10)	0.86	0.98 (0.81, 1.20)	−0.04 (−0.55, 0.46)	0.96 (0.58, 1.59)
Step 2 Model 3						
Age	0.10	(0.05)	0.06	1.11 (1.00, 1.23)	0.25 (−0.01, 0.51)	1.28 (0.99, 1.66)
Sex (0=male)	0.02	(0.14)	0.86	1.03 (0.77, 1.36)	0.02 (−0.24, 0.29)	1.02 (0.79, 1.33)
High parent, low adolescent	−**0.47**	**(0.19)**	**0.02**	**0.63 (0.43, 0.91)**	−**0.47 (**−**0.84,** −**0.09)**	**0.63 (0.43, 0.91)**
High adolescent, low parent	−**0.44**	**(0.20)**	**0.03**	**0.65 (0.43, 0.96)**	−**0.44 (**−**0.83,** −**0.04)**	**0.65 (0.43, 0.96)**
High adolescent, high parent	−**0.52**	**(0.17)**	**0.002**	**0.59 (0.43, 0.82)**	−**0.52 (**−**0.85,** −**0.19)**	**0.59 (0.43, 0.82)**

*Note. b*=unstandardized coefficients, SE=standard error, β=standardized coefficients, IRR=Incidence Rate Ratio corresponding to unstandardized coefficient.

Bolded estimates were significant.

**Table 4.** Coefficients and Incidence Rate Ratios from Hierarchical Regressions for Parental Solicitation

	*b*	SE	*p*	IRR (*b*) (95 % CI)	β (95 % CI)	IRR (β) (95 % CI)
Model 0						
Age	0.11	0.06	0.06	1.11 (1.00, 1.25)	0.27 (−0.01, 0.54)	1.31 (0.99, 1.72)
Sex (0=male)	0.05	0.15	0.72	1.05 (0.79, 1.40)	0.05 (−0.22, 0.32)	1.05 (0.80, 1.37)
Step 2 Model 1						
Age	0.09	0.06	0.12	1.09 (0.98, 1.22)	0.22 (−0.06, 0.50)	1.25 (0.94, 1.65)
Sex (0=male)	0.03	0.14	0.83	1.03 (0.78, 1.37)	0.03 (−0.24, 0.29)	1.03 (0.79, 1.34)
Adolescent Reports	−0.12	0.07	0.12	0.89 (0.77, 1.03)	−0.21 (−0.48, 0.05)	0.81 (0.62, 1.06)
Parent Reports	−0.13	0.08	0.10	0.88 (0.75, 1.03)	−0.23 (−0.50, 0.05)	0.80 (0.61, 1.05)
Step 2 Model 2						
Age	0.09	0.06	0.12	1.09 (0.98, 1.23)	0.22 (−0.05, 0.50)	1.25 (0.95, 1.65)
Sex (0=male)	0.03	0.14	0.83	1.03 (0.78, 1.37)	0.03 (−0.24, 0.30)	1.03 (0.79, 1.34)
Adolescent Reports	−0.12	0.07	0.11	0.89 (0.77, 1.03)	−0.22 (−0.48, 0.05)	0.81 (0.62, 1.05)
Parent Reports	−0.13	0.08	0.10	0.88 (0.75, 1.02)	−0.23 (−0.51, 0.04)	0.79 (0.60, 1.04)
Adolescent*Parent	−0.02	0.07	0.75	0.98 (0.84, 1.13)	−0.08 (−0.55, 0.40)	0.92 (0.57, 1.49)
Step 2 Model 3						
Age	0.08	0.06	0.19	1.08 (0.96, 1.20)	0.19 (−0.09, 0.47)	1.21 (0.91, 1.60)
Sex (0=male)	0.04	0.14	0.76	1.04 (0.79, 1.38)	0.04 (−0.22, 0.31)	1.04 (0.80, 1.36)
High parent, low adolescent	−0.27	0.19	0.15	0.76 (0.53, 1.10)	−0.27 (−0.64, 0.10)	0.76 (0.53, 1.10)
High adolescent, low parent	−0.003	0.19	0.99	1.00 (0.68, 1.45)	<.001 (−0.38, 0.37)	1.00 (0.68, 1.45)
High adolescent, high parent	−**0.44**	**0.19**	**0.02**	**0.65 (0.45, 0.93)**	**−0.44 (−.81, −0.07)**	**0.65 (0.45, 0.93)**

*Note. b*=unstandardized coefficients, SE=standard error, β=standardized coefficients, IRR=Incidence Rate Ratio corresponding to unstandardized coefficient.

Bolded estimates were significant.

**Table 5.** Coefficients and Incidence Rate Ratios from Hierarchical Regressions for Parental Control

	*b*	SE	*p*	IRR (β) (95 % CI)	β (95 % CI)	IRR (β) (95 % CI)
Model 0						
Age	0.11	0.06	0.06	1.11 (1.00, 1.25)	0.27 (−0.01, 0.54)	1.31 (0.99, 1.72)
Sex (0=male)	0.05	0.15	0.72	1.05 (0.79, 1.40)	0.05 (−0.22, 0.32)	1.05 (0.80, 1.37)
Step 2 Model 1						
Age	0.06	0.06	0.34	1.06 (0.94, 1.19)	0.14 (−0.14, 0.42)	1.15 (0.87, 1.52)
Sex (0=male)	−0.001	0.15	0.99	1.00 (0.75, 1.33)	<.001 (−0.27, 0.27)	1.00 (0.76, 1.31)
Adolescent Reports	−0.09	0.07	0.17	0.91 (0.80, 1.04)	−0.22 (−0.53, 0.09)	0.80 (0.59, 1.10)
Parent Reports	−0.13	0.09	0.16	0.87 (0.73, 1.05)	−0.21 (−0.50, 0.08)	0.81 (0.61, 1.09)
Step 2 Model 2						
Age	0.10	0.07	0.15	1.11 (0.97, 1.27)	0.25 (−0.09, 0.58)	1.28 (0.92, 1.79)
Sex (0=male)	0.16	0.18	0.39	1.17 (0.82, 1.67)	0.15 (−0.19, 0.48)	1.16 (0.83, 1.62)
Adolescent Reports	−0.13	0.08	0.07	0.87 (0.75, 1.01)	−0.32 (−0.67, 0.03)	0.73 (0.51, 1.03)
Parent Reports	−0.14	0.12	0.26	0.87 (0.68, 1.11)	−0.22 (−0.59, 0.16)	0.81 (0.55, 1.18)
Adolescent*Parent	−0.04	0.07	0.58	0.96 (0.83, 1.11)	−0.15 (−0.70, 0.39)	0.86 (0.50, 1.48)
Step 2 Model 3						
Age	0.08	0.06	0.16	1.08 (0.97, 1.21)	0.20 (−0.08, 0.47)	1.22 (0.92, 1.60)
Sex (0=male)	−0.02	0.15	0.92	0.98 (0.74 1.32)	−0.01 (−0.29, 0.26)	0.99 (0.75, 1.29)
High parent, low adolescent	−0.04	0.20	0.84	0.96 (0.64, 1.43)	−0.04 (−0.44, 0.36)	0.96 (0.64, 1.43)
High adolescent, low parent	0.06	0.24	0.81	1.06 (0.67, 1.68)	0.06 (−0.41, 0.52)	1.06 (0.67, 1.68)
High adolescent, high parent	−0.35	0.19	0.06	0.70 (0.49, 1.02)	−0.35 (−0.72, 0.02)	0.70 (0.49, 1.02)

*Note*. *b*=unstandardized coefficients, SE=standard error, β=standardized coefficients, IRR=Incidence Rate Ratio corresponding to unstandardized coefficient.

**Table 6.** Coefficients and Incidence Rate Ratios from Hierarchical Regressions for Child Disclosure

	*b*	SE	*p*	IRR (*b*) (95 % CI)	β (95 % CI)	IRR (β)(95 % CI)
Model 0						
Age	0.11	0.06	0.06	1.11 (1.00, 1.25)	0.27 (−0.01, 0.55)	1.31 (0.99, 1.73)
Sex (0=male)	0.06	0.15	0.67	1.06 (0.80, 1.42)	0.06 (−0.21, 0.33)	1.06 (0.81, 1.39)
Step 2 Model 1						
Age	**0.12**	**0.05**	**0.03**	**1.13 (1.01, 1.25)**	**0.30 (0.03, 0.56)**	**1.35 (1.03, 1.75)**
Sex (0=male)	0.02	0.14	0.87	1.02 (0.77, 1.36)	0.02 (−0.25, 0.29)	1.02 (0.78, 1.34)
Adolescent Reports	−**0.19**	**0.08**	**0.02**	**0.83 (0.71, 0.97)**	−**0.32 (**−**0.59,** −**0.06)**	**0.72 (0.55, 0.95)**
Parent Reports	−0.10	0.08	0.21	0.90 (0.76, 1.06)	−0.18 (−0.46, 0.10)	0.84 (0.63, 1.11)
						
Age	**0.12**	**0.05**	**0.03**	**1.13 (1.01, 1.26)**	**0.30 (0.03, 0.57)**	**1.35 (1.03, 1.76)**
Sex (0=male)	0.02	0.14	0.88	1.02 (0.77, 1.36)	0.02 (−0.25, 0.29)	1.02 (0.78, 1.33)
Adolescent Reports	−**0.19**	**0.08**	**0.02**	**0.82 (0.70, 0.97)**	−0.33 (−0.60, −0.06)	**0.72 (0.55, 0.95)**
Parent Reports	−0.11	0.08	0.21	0.90 (0.76, 1.06)	−0.18 (−0.46, 0.10)	0.83 (0.63, 1.11)
Adolescent*Parent	0.04	0.10	0.71	1.04 (0.86, 1.25)	0.10 (−0.44, 0.64)	1.11 (0.65, 1.90)
						
Age	**0.11**	**0.06**	**0.04**	**1.12 (1.00, 1.24)**	**0.28 (0.00, 0.55)**	**1.32 (1.00, 1.74)**
Sex (0=male)	0.04	0.15	0.81	1.03 (0.78, 1.38)	0.03 (−0.24, 0.31)	1.03 (0.79, 1.36)
High parent, low adolescent	−0.33	0.19	0.09	0.72 (0.49, 1.06)	−0.33 (−0.71, 0.05)	0.72 (0.49, 1.09)
High adolescent, low parent	−0.33	0.21	0.12	0.72 (0.47, 1.09)	−0.33 (−0.75, 0.09)	0.72 (0.47, 1.09)
High adolescent, high parent	−**0.42**	**0.17**	**0.01**	**0.66 (0.47, 0.92)**	−**0.42 (**−**0.75,** −**0.09)**	**0.66 (0.47, 0.92)**

*Note. b*=unstandardized coefficients, SE=standard error, β=standardized coefficients, IRR=Incidence Rate Ratio corresponding to unstandardized coefficient.

Bolded estimates were significant.
